# Características clínicas asociadas a niveles elevados de lipoproteína(a) en pacientes atendidos por riesgo vascular

**DOI:** 10.1515/almed-2023-0090

**Published:** 2023-10-23

**Authors:** Javier Rubio-Serrano, Alejandra Gullón Ojesto, Carmen Suárez Fernández

**Affiliations:** Fundación de Investigación Biomédica del Hospital Universitario de La Princesa, Instituto de Investigación Sanitaria Princesa, Madrid, España; Unidad de Riesgo Vascular. Servicio de Medicina Interna, Hospital Universitario de La Princesa, Madrid, España

**Keywords:** enfermedad cardiovascular, factores de riesgo, hipercolesterolemia familiar, lipoproteína(a)

## Abstract

**Objectivos:**

La lipoproteína(a) (Lp(a)) es cada vez más relevante en la evaluación de pacientes con riesgo vascular debido a su asociación con una mayor incidencia de eventos cardiovasculares. Este estudio tiene como objetivo identificar las características clínicas de los pacientes con niveles elevados de Lp(a) atendidos en consultas externas por riesgo vascular.

**Métodos:**

Estudio observacional retrospectivo en donde se compararon las características clínicas de los pacientes con niveles elevados de Lp(a) (≥50 mg/dL) con la de los pacientes con valores normales (<50 mg/dL), en un total de 878 pacientes atendidos por riesgo o enfermedad vascular durante los años 2021 y 2022.

**Resultados:**

Los valores más elevados de Lp(a) se asociaron de forma independiente con una mayor probabilidad de antecedentes de enfermedad arterial periférica (p=0,024), hipercolesterolemia familiar poligénica (HFP, p=0,030) e hipercolesterolemia familiar combinada (HFC, p=0,015), el tratamiento de inhibidores de PCSK9 (p=0,029) y la combinación de estatinas y ezetimiba (p=0,018). Sin embargo, no se obtuvieron diferencias significativas para las variables antecedentes familiares de enfermedad cardiovascular precoz (p=0,143) ni para antecedentes de enfermedad cardiovascular previa (p=0,063) a diferencia de lo identificado en otras series.

**Conclusiones:**

Los niveles elevados de Lp(a) se asociaron con antecedentes de enfermedad arterial periférica, diagnóstico de HFP y HFC, así como con la necesidad de utilizar tratamientos hipolipemiantes más intensos.

## Introducción

La lipoproteína(a) (Lp(a)) es un complejo macromolecular esférico cuya estructura, tamaño y composición lipídica se asemeja a la lipoproteína de baja densidad (LDL). La Lp(a) está compuesta por una molécula de apolipoproteína B100 (apoB100) unida covalentemente a través de un enlace disulfuro a la apolipoproteína(a) (apo(a)) [1, 2].

Las concentraciones plasmáticas de Lp(a) varían ampliamente en la población general, sin embargo, presentan una escasa variabilidad interindividual durante toda la vida [3]. Niveles elevados de Lp(a) se asocian con un mayor riesgo de enfermedad cardiovascular aterosclerótica y estenosis aórtica, especialmente en presencia de otros factores de riesgo como la hipertensión, diabetes, obesidad, tabaquismo o antecedentes familiares de enfermedad cardiovascular precoz [1, 4, 5].

Las concentraciones superiores a 30 mg/dL, y aún más por encima de 50 mg/dL, contribuyen al aumento de riesgo cardiovascular a través de diferentes tipos de mecanismos [6]. En primer lugar, la Lp(a) promueve el desarrollo de placas de ateroma como consecuencia de la unión de la apo(a) a la proteína plasmática plasmina del revestimiento de la arteria [7]. Además, la Lp(a) participa en la activación de las vías de señalización proinflamatorias y en la disminución de la disponibilidad de óxido nítrico endotelial [8, 9]. Por otra parte, la Lp(a) tiene la capacidad de interferir con la fibrinólisis al unirse a la plasmina y, de este modo, reducir su actividad para disolver los coágulos en la superficie interna de los vasos sanguíneos. Esta interferencia puede aumentar el riesgo de formación de trombos y la obstrucción arterial, contribuyendo al desarrollo de enfermedades cardiovasculares [10].

Investigaciones previas han demostrado una mayor prevalencia de Lp(a) elevada en pacientes con hipercolesterolemia familiar (HF), un trastorno genético hereditario caracterizado por niveles elevados de colesterol LDL (cLDL), aumentado significativamente el riesgo de enfermedad cardiovascular ateroesclerótica [11, 12]. Este trastorno metabólico está causado principalmente por mutaciones patogénicas en genes que codifican para el receptor de LDL (RLDL) [13, 14], la proproteína convertasa subtilisina kexina 9 (PCSK9) [14, 15] o la apoliproteína B (APOB) [14, 16].

A pesar de que en la actualidad no existen tratamientos farmacológicos aprobados específicamente para disminuir los niveles de Lp(a), su determinación en pacientes con riesgo vascular puede contribuir a estratificar su riesgo y por lo tanto modificar sus objetivos terapéuticos [17]. Según las recomendaciones de la Sociedad Española de Arteriosclerosis (SEA), se sugiere realizar al menos una vez en la vida la solicitud de la medición de la Lp(a) en pacientes evaluados por riesgo vascular, debido a su importante consideración como un marcador pronóstico potente [18]. El objetivo principal de este estudio fue la caracterización clínica de pacientes con niveles elevados de Lp(a), atendidos en consultas externas por riesgo o enfermedad vascular.

## Materiales y métodos

### Tipo de estudio

Se realizó un estudio observacional retrospectivo en pacientes atendidos por riesgo vascular en consultas externas del Hospital Universitario de La Princesa, centro hospitalario de tercer nivel localizado en Madrid que cubre la asistencia sanitaria de un área poblacional de 323.000 personas. La inclusión de pacientes se realizó durante dos años consecutivos, 2021 y 2022.

### Selección de pacientes

La selección de la muestra se realizó mediante muestreo consecutivo de todos los pacientes a los que se determinó los niveles de Lp(a) en sangre, atendidos por riesgo vascular (con o sin antecedente de evento cardiovascular) en las consultas externas de Medicina interna, Cardiología, Endocrinología y Neurología durante los años 2021 y 2022. Se incluyeron un total de 878 pacientes que cumplieron con dicho criterio de inclusión.

Este estudio fue aprobado por el Comité Ético de Investigación Clínica del Hospital Universitario de La Princesa y en todo momento se garantizó la confidencialidad de los datos personales de los pacientes.

### Variables de estudio

El conjunto de variables clínicas y analíticas recogidas rutinariamente durante las visitas de los pacientes fueron las siguientes: demográficas (edad y sexo); servicio responsable de la solicitud de los niveles de Lp(a); factores de riesgo vascular (antecedentes de dislipemia, hipertensión, diabetes, tabaquismo, obesidad y antecedentes familiares de enfermedad cardiovascular precoz); antecedentes de enfermedad cardiovascular previa (cardiopatía isquémica, enfermedad cerebrovascular, estenosis aórtica y enfermedad arterial periférica); diagnóstico de dislipemia familiar (diferenciando entre hipercolesterolemia familiar heterocigota (HFH), hipercolesterolemia familiar poligénica (HFP) e hipercolesterolemia familiar combinada (HFC)); niveles de Lp(a) (mg/dL) y cLDL (mg/dL) en sangre; y finalmente los tratamientos hipolipemiantes utilizados: estatinas de alta potencia (atorvastatina ≥40 mg o rosuvastatina ≥10 mg), estatinas de baja potencia (atorvastatina <40 mg, rosuvastatina <10 mg, simvastatina, pravastatina, lovastatina, fluvastatina o pitavastatina), ezetimiba y/o inhibidores de PCSK9 (iPCSK9).

Las técnicas actuales de laboratorio para la determinación de Lp(a) pueden sufrir variabilidad en sus resultados debido a la heterogeneidad del tamaño de molécula de apo(a) condicionada según el número de dominios kringle 4 de tipo 2 del gen que la codifica. Teniendo en cuenta esto, la determinación cuantitativa de Lp(a) en nuestro centro se realizó mediante técnicas inmunoturbidimétricas con sistemas Roche/Hitachi cobas c 701/702 con anticuerpos dirigidos a la apo(a), al ser técnicas de cuantificación estandarizadas contra un método independiente del tamaño de apo(a). Los niveles se obtuvieron en nmol/L y, posteriormente, se realizó la conversión a mg/dL para su interpretación clínica.

### Análisis estadístico

Según la literatura, las concentraciones plasmáticas óptimas se corresponden con valores inferiores a 30 mg/dL, sin embargo, en la práctica clínica se ha observado una clara asociación con un mayor riesgo de enfermedad cardiovascular en niveles de Lp(a) superiores a 50 mg/dL [19], [20], [21]. Por consiguiente, se compararon las características clínicas de los pacientes con niveles elevados de Lp(a) (Lp(a)≥50 mg/dL) frente a aquellos con valores asociados a un menor riesgo de enfermedad cardiovascular (Lp(a)<50 mg/dL).

Se calcularon las medidas de tendencia central como la media y la desviación estándar para las variables cuantitativas, mientras que los datos cualitativos se expresaron como frecuencias y porcentajes absolutos.

En el análisis univariante, debido a la distribución no paramétrica de los datos, se utilizó la prueba de Mann-Whitney U para determinar las diferencias entre las variables categóricas y las variables continuas. Sin embargo, cuando ambas variables fueron categóricas se utilizó el test de chi-cuadrado (χ^2^). En el modelo multivariante se incluyeron las variables independientes cuyo p-valor fue inferior a 0,1 en el análisis univariante, así como aquellas que tenían una importancia clínica asociada a los valores de Lp(a) según la literatura [4]. Para el modelo multivariante se utilizó el modelo de regresión logística por pasos hacia atrás. Se estableció la significación estadística cuando el error alfa fue inferior a 0,05 con un intervalo de confianza del 95 %. Para la realización de todos los análisis estadísticos se empleó el software informático R versión 4.3.0 [22].

## Resultados

Se incluyeron un total de 878 pacientes atendidos por riesgo vascular durante los años 2021 y 2022, con determinación de los niveles de Lp(a) en sangre. Los servicios que solicitaron la determinación de la Lp(a) fueron Medicina interna (48 %), Cardiología (46 %), Endocrinología (2 %), Neurología (1 %) y otros servicios (3 %). De la totalidad de la muestra, 274 pacientes (27,2 %) presentaron niveles elevados de Lp(a) (Lp(a)≥50 mg/dL). Entre los pacientes con Lp(a) elevada, 17 (1,9 %) presentaron niveles de Lp(a)≥180 mg/dL que han sido relacionados a un riesgo a lo largo de la vida de evento vascular similar al asociado a una HFH [23]. Además, 84 pacientes (10,7 %) obtuvieron niveles entre 30 y 50 mg/dL y los 520 pacientes restantes (62,1 %) presentaron valores inferiores a 30 mg/dL, considerados niveles normales en la población general [24].

En cuanto a las características basales, la cohorte de estudio presentó una edad media de 61 años y fue representada en un 37 % por mujeres. Atendiendo a los factores de riesgo vascular, más de la mitad de la población presentó dislipemia e hipertensión, la mayoría de los pacientes tenían un índice de masa corporal (IMC) compatible con sobrepeso (27,3 %) y solo un 28,4 % de los pacientes tuvo antecedentes familiares de enfermedad cardiovascular precoz. Más de la mitad de la muestra había presentado un evento previo de enfermedad vascular, siendo el más relevante la cardiopatía isquémica (48,2 %), mientras que la enfermedad cerebrovascular (7,4 %), la enfermedad arterial periférica (6,3 %) y la estenosis aórtica (2,3 %) presentaron una incidencia inferior. El 15,9 % de los pacientes presentaban antecedente de dislipemia familiar, siendo el diagnóstico de hipercolesterolemia familiar poligénica el más frecuente. Por último, teniendo en cuenta los tratamientos hipolipemiantes empleados, más de la mitad de la población de estudio tomaba estatinas de alta potencia (53,4 %), estatinas + ezetimiba (33,1 %), estatinas + iPCSK9 (2,8 %) y estatinas + ezetimiba + iPCSK9 (1,7 %) (Tabla 1).

**Tabla 1:** Variables clínicas y antropométricas de la población de estudio.

Variables	Población total (n=878)	Lp(a)<50 mg/dL (n=604)	Lp(a)≥50 mg/dL (n=274)
Edad, años, media ± DE	60,8 ± 14,0	60,4 ± 13,9	61,8 ± 14,2
Sexo, mujer, n (%)	321 (36,6)	209 (34,6)	112 (40,9)
Factores de riesgo vascular			
Dislipemia, n (%)	674 (76,8)	449 (74,3)	225 (82,1)
cLDL, mg/dL, media ± DE	90,2 ± 40,1	90,7 ± 40,8	89,1 ± 38,5
Hipertensión, n (%)	516 (58,8)	361 (59,8)	155 (56,6)
Diabetes, n (%)	202 (23,0)	136 (22,5)	66 (24,1)
Tabaquismo activo, n (%)	212 (24,1)	160 (26,7)	52 (19,1)
IMC, media ± DE	27,3 ± 4,7	27,5 ± 4,8	26,9 ± 4,6
AF de ECVP, n (%)^a^	129 (28,4)	82 (26,6)	47 (32,0)
Enfermedad cardiovascular previa			
ECV total, n (%)	479 (54,6)	329 (54,5)	150 (54,7)
Cardiopatía isquémica, n (%)	423 (48,2)	291 (48,2)	132 (48,2)
Enfermedad cerebrovascular, n (%)	65 (7,4)	48 (8,0)	17 (6,2)
Estenosis aórtica, n (%)	20 (2,3)	14 (2,3)	6 (2,2)
EAP, n (%)	55 (6,3)	30 (5,0)	25 (9,1)
Dislipemia familiar			
HFH, n (%)	28 (3,2)	15 (2,5)	13 (4,7)
HFP, n (%)	93 (10,6)	50 (8,3)	43 (15,7)
HFC, n (%)	18 (2,1)	8 (1,3)	10 (3,7)
Tratamientos hipolipemiantes			
Estatinas de alta potencia, n (%)	469 (53,4)	310 (51,3)	159 (58,0)
Estatinas de baja potencia, n (%)	179 (20,4)	116 (19,2)	63 (23,0)
Ezetimiba, n (%)	326 (37,1)	195 (32,3)	131 (47,8)
iPCSK9, n (%)	47 (5,4)	20 (3,3)	27 (9,9)
Estatinas + ezetimiba, n (%)	291 (33,1)	172 (28,5)	119 (43,4)
Estatinas + iPCSK9, n (%)	25 (2,8)	10 (1,7)	15 (5,5)
Estatinas + ezetimiba + iPCSK9, n (%)	15 (1,7)	4 (0,7)	11 (4,0)

DE, desviación estándar; cLDL, colesterol de lipoproteínas de baja densidad; IMC, índice de masa corporal; AF de ECVP, antecedentes familiares de enfermedad cardiovascular precoz; ECV, enfermedad cardiovascular; EAP, enfermedad arterial periférica; HFH, hipercolesterolemia familiar heterocigota; HFP, hipercolesterolemia familiar poligénica; HFC, hipercolesterolemia familiar combinada; iPCSK9, inhibidores de la proteína convertasa subtilisina kexina tipo 9. ^a^423 valores faltantes; el tamaño muestral fue de 455 pacientes, 147 presentaron niveles elevados de Lp(a) mientras que 308 pacientes presentaron niveles normales.

En el análisis univariante, se relacionaron los niveles elevados de Lp(a) con los factores de riesgo dislipemia y con menor probabilidad de tabaquismo activo. En cuanto al resto de factores, incluida la variable antecedentes familiares de enfermedad cardiovascular precoz, no se obtuvieron diferencias significativas entre ambas poblaciones de estudio. Respecto a los antecedentes de enfermedad vascular previa, los niveles elevados de Lp(a) se relacionaron con el antecedente de enfermedad arterial periférica. Sin embargo, no se observaron relaciones significativas con el resto de eventos cardiovasculares (Tablas 1 y 2).

**Tabla 2:** Análisis univariante y multivariante.

Variables	Univariante		Multivariante	
	Odds ratio	p	Odds ratio ajustado	p
Edad, años	–	0,167	–	–
Sexo (mujer)	0,8 (0,6–1,0)	0,087	–	–
Factores de riesgo vascular				
Dislipemia	1,6 (1,1–2,3)	0,015^a^	1,2 (0,7–2,3)	0,486
cLDL, mg/dL	–	0,674	–	–
Hipertensión	0,9 (0,7–1,2)	0,413	–	–
Diabetes	1,1 (0,8–1,5)	0,670	–	–
Tabaquismo activo	0,7 (0,5–0,9)	0,019^a^	0,6 (0,3–1,0)	0,080
IMC	–	0,100	–	–
AF de ECVP	1,3 (0,8–2,0)	0,283	1,4 (0,9–2,3)	0,143
Enfermedad cardiovascular previa				
ECV total	1,0 (0,8–1,4)	0,998	0,3 (0,1–1,0)	0,063
Cardiopatía isquémica	1,0 (0,8–1,3)	1,000	2,4 (0,7–9,0)	0,167
Enfermedad cerebrovascular	0,8 (0,2–1,3)	0,439	–	–
Estenosis aórtica	1,0 (0,3–2,4)	1,000	–	0,986
EAP	1,9 (1,1–3,3)	0,027^a^	3,4 (1,2–10,4)	0,024^a^
Dislipemia familiar				
HFH	2,0 (0,9–4,2)	0,119	0,9 (0,3–2,5)	0,814
HFP	2,1 (1,3–3,2)	0,001^b^	1,9 (1,1–3,5)	0,030^a^
HFC	2,8 (1,1–7,5)	0,046^a^	3,7 (1,3–11,1)	0,015^a^
Tratamientos hipolipemiantes				
Estatinas de alta potencia	1,3 (1,0–1,8)	0,076	–	–
Estatinas de baja potencia	1,3 (0,9–1,8)	0,230	–	–
Ezetimiba	1,9 (1,4–2,6)	<0,001^c^	0,6 (0,2–1,7)	0,360
iPCSK9	3,2 (1,8–5,9)	<0,001^c^	5,0 (1,2–22,9)	0,029^a^
Estatinas + ezetimiba	1,9 (1,4–2,6)	<0,001^c^	4,4 (1,4–1,6)	0,018^a^
Estatinas + iPCSK9	3,4 (1,5–8,0)	0,003^b^	1,2 (0,1–1,3)	0,884
Estatinas + ezetimiba + iPCSK9	6,1 (2,0–22,9)	0,001^b^	0,5 (0,0–5,8)	0,580

cLDL, colesterol de lipoproteínas de baja densidad; IMC, índice de masa corporal; AF de ECVP, antecedentes familiares de enfermedad cardiovascular precoz; ECV, enfermedad cardiovascular; EAP, enfermedad arterial periférica; HFH, hipercolesterolemia familiar heterocigota; HFP, hipercolesterolemia familiar poligénica; HFC, hipercolesterolemia familiar combinada; iPCSK9, inhibidores de la proteína convertasa subtilisina kexina tipo 9. ^a^p<0,05; ^b^p<0,01; ^c^p<0,001.

El porcentaje de pacientes con diagnóstico de HFP y HFC fue mayor en grupo de pacientes con niveles elevados de Lp(a) (15,7 y 3,7 % respectivamente). Sin embargo, la prevalencia de HFH fue similar entre ambas poblaciones de estudio.

En cuanto a los tratamientos hipolipemiantes empleados, el uso de ezetimiba e iPCSK9 fue significativamente superior en el subgrupo de Lp(a) más elevada: 47,8 vs. 32,3 % para ezetimiba y 9,9 vs. 3,3 % para iPCSK9. Del mismo modo, la combinación de fármacos hipolipemiantes fue significativamente superior en el grupo de pacientes con niveles elevados de Lp(a): 43,4 vs. 28,5 % para el uso de estatinas + ezetimiba, 5,5 vs. 1,7 % para estatinas + iPCSK9 y 4,0 vs. 0,7 % para estatinas + ezetimiba + iPCSK9 (Tablas 1 y 2).

Finalmente, las variables con un p-valor inferior a 0,05 en el análisis univariante, así como aquellas que tenían una importancia clínica según la literatura (antecedentes familiares de enfermedad cardiovascular precoz, enfermedad cardiovascular previa, cardiopatía isquémica, estenosis aórtica y HFH) se incluyeron en un modelo multivariante de regresión logística. Estos análisis confirmaron la relación independiente de los niveles de Lp(a) elevados con la enfermedad arterial periférica, el diagnóstico de HFP y HFC y el tratamiento con iPCSK9 y la combinación de estatinas + ezetimiba (Tabla 2).

## Discusión

La determinación de niveles elevados de Lp(a) se han relacionado con un mayor riesgo de enfermedad vascular, cardiopatía isquémica precoz y estenosis aórtica [4, 5]. En la actualidad, no existen tratamientos farmacológicos aprobados específicamente para la disminución de los niveles de Lp(a). Sin embargo, la caracterización clínica de estos pacientes puede ayudar a la reclasificación de su riesgo vascular, favoreciendo la adecuación de las medidas y objetivos terapéuticos con intención de disminuir la aparición de complicaciones cardiovasculares ateroscleróticas [25].

En el presente estudio, se evaluó las características clínicas relacionadas con la presencia de niveles elevados de Lp(a) en 878 pacientes atendidos por riesgo vascular en un hospital terciario de la comunidad autónoma de Madrid. En la muestra, los niveles de Lp(a)≥a 50 mg/dL se asociaron a pacientes con antecedentes personales de dislipemia y con una menor prevalencia de tabaquismo activo. Por el contrario, no se obtuvieron diferencias significativas en la variable antecedentes familiares de enfermedad cardiovascular precoz, en contraste con los hallazgos del estudio de Quyyumi et al. [26], donde observaron una asociación conjunta de los antecedentes familiares de enfermedad cardiovascular y niveles elevados de Lp(a) con el riesgo cardiovascular a largo plazo.

En nuestro estudio, los niveles elevados de Lp(a) se asociaron con el antecedente de enfermedad arterial periférica, sin embargo, no se observaron diferencias en cuanto a la prevalencia del resto de antecedentes de enfermedad cardiovascular. Una posible justificación de la falta de diferencias significativas podría ser el hecho de que los pacientes incluidos en la muestra fueron atendidos en consultas específicas por riesgo vascular, lo que puede haber generado un sesgo de selección y haber influenciado los resultados. De hecho, más de la mitad de la población total del estudio estaba en prevención secundaria (pacientes que presentaron un evento previo: ictus, cardiopatía isquémica o arteriopatía periférica).

Además, los pacientes con diagnóstico de HFP y HFC se asociaron con niveles más elevados de Lp(a), siendo el número de pacientes con dicho diagnóstico dos y cuatro veces superior, respectivamente, al grupo de pacientes con niveles normales. Aunque no se observaron diferencias estadísticamente significativas para la HFH, se podría asumir que la determinación de niveles elevados de Lp(a) se asocia con el diagnóstico de hiperlipemias familiares, tal y como se ha reportado en literatura previa [27].

La presencia de unos valores elevados de Lp(a) no se correlacionó con una mayor prescripción de estatinas de alta potencia, si bien el porcentaje de pacientes tratados con estos fármacos fue elevado en ambos grupos (>50 %), de acuerdo con el porcentaje elevado de antecedente de evento vascular previo. Los pacientes con Lp(a) elevada recibían con mayor frecuencia una terapia hipolipemiante más intensiva (mayor uso combinado de ezetimiba asociado a estatinas e iPCSK9). Este hecho sugiere una intencionalidad por parte de los médicos de alcanzar un objetivo de cLDL más bajo por ser pacientes de más alto riesgo, siguiendo las recomendaciones de las guías de práctica clínica de la Sociedad Europea de Cardiología (ESC), y también puede sugerir la posibilidad de buscar objetivos terapéuticos más exigentes en pacientes con niveles de Lp(a) elevados, considerando su alto riesgo vascular [28] y la ausencia de terapias específicas aprobadas para descender la Lp(a). Ensayos clínicos actuales han demostrado la capacidad de los iPCSK9 para disminuir sinérgicamente los niveles de cLDL y Lp(a) [29, 30], reportándose reducciones del 55 y 25 % respectivamente [31, 32]. El beneficio clínico atribuible a la reducción de estos niveles aún no ha sido claramente demostrado en la literatura, pero las tendencias parecen sugerir su eficacia en la reducción de los eventos cardiovasculares mayores [33].

Nuestro estudio presenta varias limitaciones. Su carácter retrospectivo limitó el acceso a algunas de las variables como fue el caso del antecedente familiar de enfermedad cardiovascular precoz que tuvo 423 datos faltantes, siendo probablemente la explicación por la que no se obtuvieron diferencias significativas en esta variable entre las poblaciones del estudio. Además, su diseño observacional no permite establecer relaciones de causalidad sino sólo hipótesis asociativas. Los resultados obtenidos no permiten identificar características clínicas notables que diferencien claramente a los pacientes con niveles elevados de Lp(a) dentro del espectro de pacientes atendidos por riesgo vascular. Sin embargo, el elevado tamaño muestral y la heterogeneidad de los pacientes incluidos, nos permite dar respuesta al objetivo planteado.

En conclusión, en nuestra muestra de pacientes atendidos por riesgo vascular en un hospital terciario, los niveles elevados de Lp(a) se relacionaron con el diagnóstico de enfermedad arterial periférica, de HFP y HFC, así como con el uso de los tratamientos hipolipemiantes de mayor intensidad.
